# Diagnostic accuracy of quantitative light-induced fluorescence in detecting caries of various types and locations: a systematic review and meta-analysis

**DOI:** 10.1038/s41598-025-23631-6

**Published:** 2025-11-14

**Authors:** Eun-Song Lee, Jeehyun Hwang, Lei Cheng, Hoi In Jung, Baek-Il Kim

**Affiliations:** 1 Department of Preventive Dentistry and Public Oral Health, BK21 FOUR Project, Yonsei University College of Dentistry, 50-1 Yonsei-ro, Seodaemun-gu, Seoul, 03722 Republic of Korea; 2 State Key Laboratory of Oral Diseases and National Center for Stomatology and National Clinical Research Center for Oral Diseases, West China Hospital of Stomatology, Sichuan University, Chengdu, 610041 China

**Keywords:** Quantitative light-induced fluorescence, Dental caries, Diagnostic accuracy, Systematic review, Meta-analysis, Biomarkers, Diseases, Health care, Medical research

## Abstract

This systematic review and meta-analysis aimed to assess the diagnostic accuracy of quantitative light-induced fluorescence (QLF) in detecting dental caries of varying lesion severities, surfaces, and dentition types, across both in vitro and in vivo studies. Data extracted included study characteristics, diagnostic outcomes (sensitivity, specificity, AUC), caries types, lesion thresholds (ICDAS), and QLF parameters (ΔF, ΔR). Enamel lesions were stratified into incipient (ICDAS 1–2) and advanced stages (ICDAS 3), with dentin caries defined as ICDAS 4 or greater. A comprehensive search of Ovid MEDLINE and EMBASE was conducted up to May 2023, without language restrictions. Manual reference screening was performed to identify additional relevant studies. Seventeen studies met the eligibility criteria, which included evaluating QLF against reference standards (ICDAS, radiography, histology) and reporting diagnostic measures. Risk of bias was assessed using QUADAS-2. Meta-analysis included studies with extractable or inferable 2 × 2 contingency data. QLF demonstrated excellent diagnostic accuracy in distinguishing sound surfaces from enamel and dentin lesions, with in vivo Area Under the Curve (AUC) values for incipient occlusal lesions ranging from 0.94 to 0.98. The technology showed high pooled sensitivity and specificity for both occlusal (in vivo: 0.86/0.82) and approximal caries (in vivo: 0.74/0.82), confirming its effectiveness for early-stage detection.

## Introduction

Dental caries affects approximately 3.09 billion people worldwide and is the leading cause of global disease burden, making it one of the most prevalent chronic diseases^1^. To address this urgent public health issue, it is essential to understand the pathophysiology and progression of dental caries and develop technologies capable of the early detection of lesions at the initial stages, where non-surgical management is possible, or accurate diagnosis of advanced lesions requiring surgical intervention.

Recent research on caries ecology has introduced the concept of a dynamic balance between demineralisation and remineralisation driven by changes in the oral microbial ecosystem, emphasising the importance of lesion activity assessment and stage-specific disease management^2,3^. However, traditional diagnostic methods, such as visual-tactile examination and radiographic assessment, which are effective for advanced carious lesions, have significant limitations with markedly low sensitivity for detecting subtle initial enamel demineralisation lesions, making early diagnosis challenging^4,5^. Recently, the latest ORCA-EFCD consensus report acknowledges the limitations of relying solely on visual examination and states that adjunct, non-ionising radiation methods might be useful in certain clinical situations to supplement visual examination and improve diagnostic accuracy^6^.

Fluorescence-based methods have been employed for the early detection and diagnosis of dental caries, supporting visual examinations in clinical settings^7^. These methods utilise light sources of different wavelengths and quantify the resulting fluorescence response based on various optical principles^7,8^. One notable technology is quantitative light-induced fluorescence (QLF), which enables simultaneous detection and quantification of biofluorescence from hard dental tissues and bacterial biofilms^9,10^. The QLF is based on two primary diagnostic principles. First, carious lesions exhibit decreased mineral content compared with sound tooth structures, leading to increased light scattering and a corresponding reduction in natural fluorescence intensity. This loss of fluorescence is quantified as delta F (ΔF)^9,11^. Second, QLF captures the red fluorescence emitted by bacterial metabolites, such as porphyrins, which are prevalent in oral biofilms and carious lesions^9,12,13^. Red fluorescence gain was quantified as delta R (ΔR). To apply both principles, QLF employs visible light at a wavelength of 405 nm along with specialised filters, enabling non-invasive and automatic acquisition of fluorescence images^11^. These images are analysed using proprietary software to quantify fluorescence loss and red fluorescence gain^14,15^. This dual-parameter approach allows for real-time assessment of caries severity and facilitates the monitoring of subtle changes in lesions^14,16^. Furthermore, real-time visualisation of diagnostic results, digital image documentation, and storage enable continuous monitoring and improve communication and motivation among patients.

Despite the growing interest in QLF technology, previous reviews on its diagnostic performance for dental caries have been limited in scope, often including only a few studies or focusing exclusively on laboratory data^7,17,18^. These earlier attempts lacked conclusive evidence, particularly regarding clinical applicability, and failed to comprehensively assess the diagnostic accuracy of the QLF across different caries types, lesion severities, and tooth locations. Furthermore, considerable heterogeneity in diagnostic performance among studies has been reported, which may be attributable to variations in reference standards, lesion definitions, and device versions^7,18^. These gaps highlight the need for an updated and methodologically rigorous evidence synthesis.

Therefore, the objective of this systematic review and meta-analysis was to comprehensively evaluate and synthesize the evidence on the diagnostic accuracy of QLF for detecting dental caries. We assessed its performance across different study settings (in vivo and in vitro), lesion locations (occlusal and proximal), dentition types, and caries depths by comparing QLF outcomes with established reference standards.

## Results

### Study selection and inclusion results

**Fig. 1:**
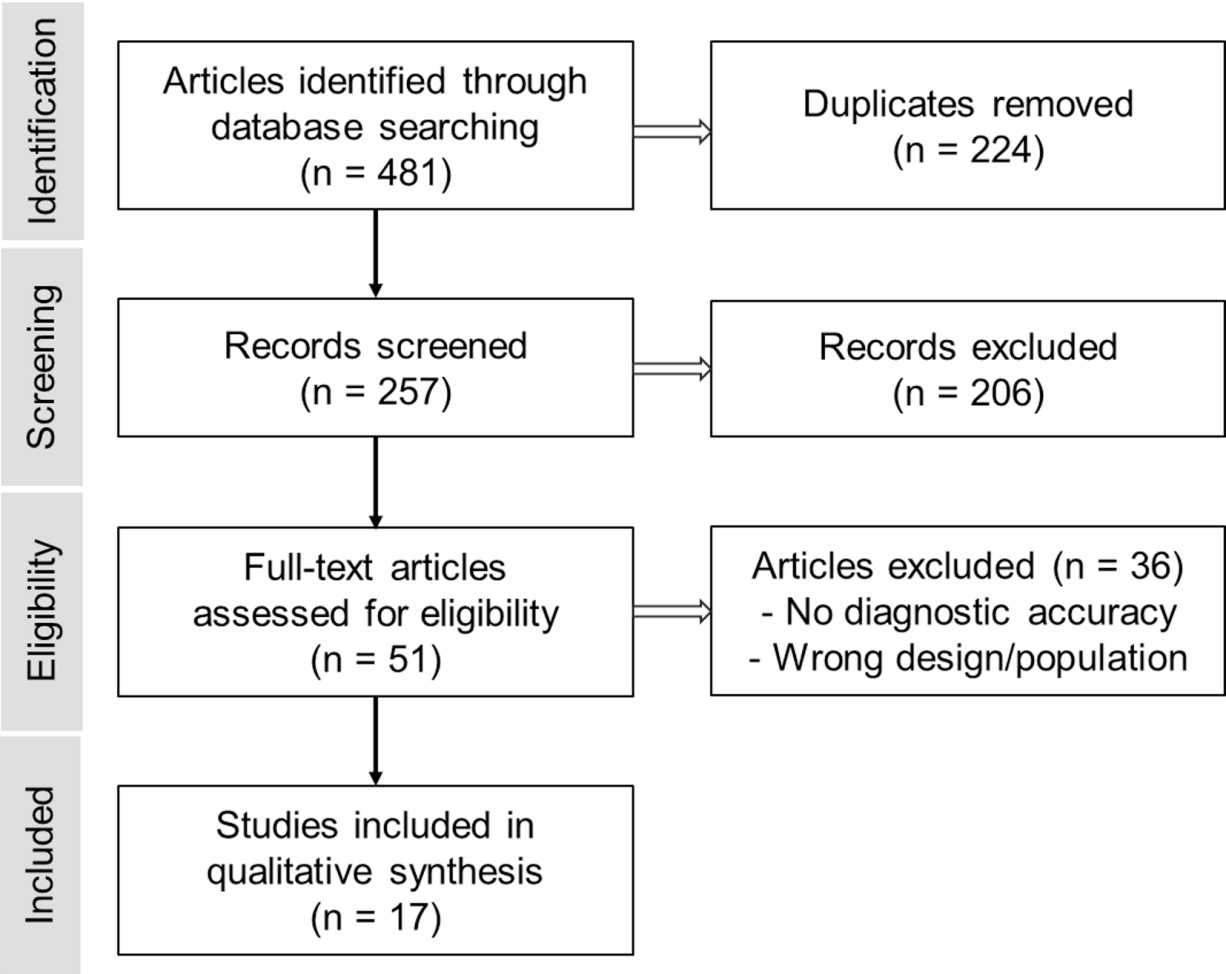
Flow diagram of the study selection process.

A total of 481 articles were initially identified through database searches (242 from PubMed and 239 from Embase). After removing 224 duplicates, 257 articles remained for screening. Title and abstract screening excluded 206 articles, and a full-text review led to the exclusion of 36 studies that did not meet the eligibility criteria. Ultimately, 17 articles met the inclusion criteria and were included in the systematic review; of these, a subset was used for the meta-analysis (Fig. 1).

### Study characteristics and methodological quality

A detailed summary of the included study characteristics is provided in Table 1. Of the 17 included studies, 5 were conducted in vivo, 11 in vitro and 1 study^19^ utilized a mixed design, where QLF measurements were performed in vivo on patients, followed by histological validation in vitro on the same teeth after extraction. In vivo studies involved sample sizes ranging from 40 to 627 teeth, whereas in vitro studies evaluated between 21 and 400 teeth. Four studies^19–22^ assessed caries in primary dentition, while the remaining focused on permanent dentition. Most studies evaluated primary caries, although three studies^22–24^ also included secondary carious lesions.

Regarding lesion location, occlusal surfaces were the most frequently evaluated (11 studies), followed by proximal surfaces (five studies), with one study focusing on smooth surfaces. Four types of QLF devices were used across the studies: QLF-Clin, Inspektor Pro, QLF-D, and Qraypen C. The most recent in vivo studies utilised the intraoral camera-type Qraypen C, whereas the DSLR type QLF-D was commonly used in in vitro studies post-2015.

**Table 1:** List of selected studies.

Ref.	First Author (year)	*n*	In vivo /In vitro	Dentition	Surface	Index test	Comparator	Reference test	Examiners	Index test Observer Reliability(ICC)
1	Oh, Choi and Kim (2022)^23^	178	In vivo	Permanent	· Occlusal · Proximal · Secondary^4)^	Qraypen C	na	ICDAS II Radiograph^3)^	1	Intra: 0.84–0.91 Inter: 0.58− 0.75
2	Cho (2021)^20^	878	In vivo	Primary	· Occlusal · Proximal	Qraypen C	na	Radiograph ICDAS II	2	Intra: 0.80^*^ Inter: 0.82^*^
3	Oh, Choi and Kim (2021)^25^	297	In vivo	Permanent	· Occlusal · Proximal	Qraypen C	na	ADA classification Bitewing	1	Intra: >0.90
4	Diniz (2019)^21^	88	In vitro	Primary	· Occlusal	Inspektor Pro	ICDAS DIAGNOdent	Histology	1	Intra: 0.92 Inter: na
5	Jung (2018)^26^	791	In vivo	Permanent	· Occlusal	QLF-D	DIAGNOdent	ICDAS II	1	Intra: 0.94 Inter: 0.86
6	Lee (2018)^27^	62	In vitro	Permanent	· Occlusal	QLF-D	na	Histology	1	na
7	Kim (2017)^28^	280	In vivo	Permanent	· Proximal	QLF-D	na	Radiograph	1	Intra: 0.72 Inter: na
8	Pontes (2017)^19^	50	In vivo /in vitro	Primary	· Occlusal	Inspektor Pro	ICDAS DIAGNOdent	Histology	1	na
9	Yoon (2017)^29^	102	In vitro	Permanent	· Proximal	QLF-D	modified ICDAS II	Radiograph	1	Intra: 0.84 Inter: na
10	Diniz (2016)^24^	180	In vitro	Permanent	· Secondary · Occlusal^1)2)^	QLF QLF-Clin	ICDAS DIAGNOdent	Histology	2	Intra: 0.92–0.93 Inter: 0.87–0.90
11	Lenzi (2016)^22^	42	In vitro	Primary	· Secondary · Occlusal^1)^	QLF QLF-Clin	ICDAS Radiograph	Histology	2	Intra: 0.91-97 Inter: 0.95
12	Jallad (2015)^30^	60	In vitro	Permanent	· Occlusal	QLF-D	ICDAS	Histology	3	Intra: 0.96–0.99 Inter: 0.96
13	Ko (2015)^31^	95	In vitro	Permanent	· Proximal	QLF-D	ICDAS Radiograph	Histology	1	Intra: 0.78 Inter: na
14	Gomez (2013)^32^	112	In vitro	Permanent	· Occlusal	Inspektor Pro	ICDAS	Histology	1	na
15	Pereira, Eggertsson, González-Cabezas, Zero, Eckert and Mialhe (2011)^33^	96	In vitro	Permanent	· Occlusal	Inspektor Pro	Ekstrand score Bitewing DIAGNOdent	Histology	3	Intra: 0.77–0.87 Inter: 0.50–86
16	Zandoná, Santiago, Eckert, Fontana, Ando and Zero (2010)^34^	400	In vivo	Permanent	· Occlusal	Inspektor Pro	na	ICDAS	1	na
17	Shi, Tranaeus, Fau and Angmar-Månsson (2001)^35^	40	In vitro	Permanent	· Smooth	Inspektor Pro	DIAGNOdent	Histology	na	na

na, not available; n, number of teeth; ICC, intraclass correlation coefficient; E, enamel caries; D, dentin caries.

^1)^Resin restoration.

^2)^Amalgam restoration.

^3)^For suspected proximal caries only.

^4)^Surface and material not available.

^*^Cohen’s kappa coefficient.

**Fig. 2:**
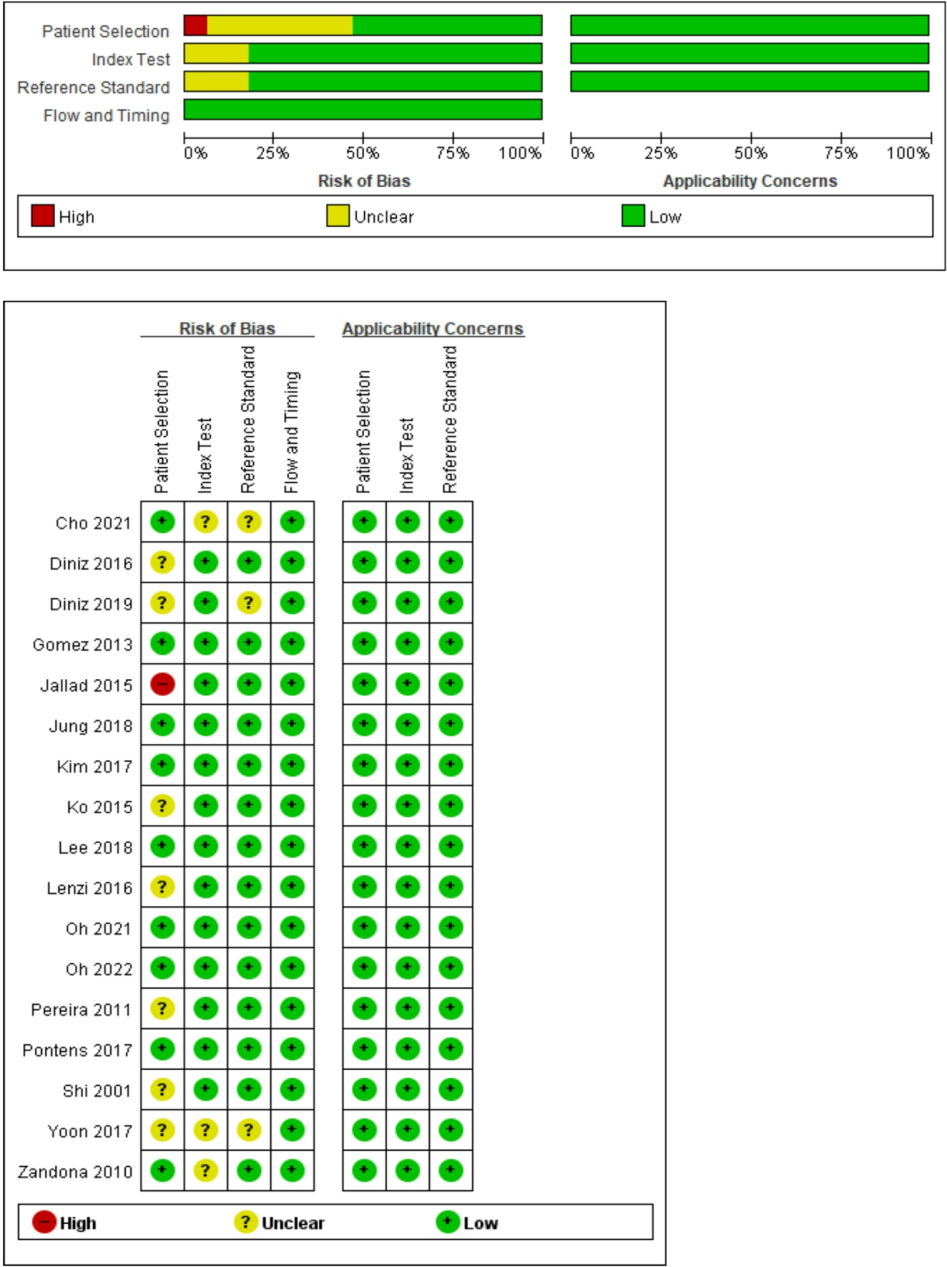
Results of the risk of bias assessment per study, according to the quality assessment of the diagnostic accuracy of studies (QUADAS-2).

The risk of bias analysis using the QUADAS-2 checklist is shown in Fig. 2. In most studies, the conduct of the index test preceded the reference test and was examined separately. Most articles were of good quality, and some demonstrated differences. Most studies used an acceptable reference standard. Eight studies did not report relevant clinical information. Bias in the blinding of the reference standard or index test results and sample selection was the most likely form of bias in the selected studies. Regarding applicability concerns, both the index and reference tests matched the expectations of our main question.

### Qualitative and quantitative analysis

Four in vivo studies focused on the detection of occlusal and proximal caries. In the detection of incipient versus advanced enamel occlusal lesions, the range of sensitivity was 0.76–0.91, the range of specificity was 0.74–0.93, and the range of the AUC was 0.81–0.93. For the sound teeth and enamel versus dentinal lesion threshold, the range of sensitivity was 0.90–0.98, the range of specificity was 0.83–0.96, and the range of the AUC was 0.94–0.98 (Table 2). In the detection of enamel caries versus dentin caries on proximal surfaces, the range of sensitivity was 0.63–0.91, the range of specificity was 0.62–0.74, and the range of the AUC was 0.67–0.91.

Six in vitro studies detected occlusal caries, three proximal caries, and one smooth surface caries. In detecting sound teeth versus enamel and dentinal occlusal lesions, the range of sensitivity was 0.68–0.96, the range of specificity was 0.75–0.92, and the range of the AUC was 0.71–0.97. For the sound teeth versus enamel and dentinal proximal threshold, the range of sensitivity was 0.75–0.80, the range of specificity was 0.84–0.92, and the range of the AUC was 0.80–0.91 (Table 3).

**Table 2:** In vivo results.

Ref.	Author (year)	*n*	Dentition	Reference test	Diagnostic accuracy				
					Diagnostic threshold (ICDAS code)	Cut-off	Se	Sp	AUC
*Occlusal*									
1	Oh (2022)	130	Permanent	ICDAS II	S vs. E (0 vs. 1–3)	ΔF: 12.9	0.89	0.75	0.92
					S, IE vs. AE (0–2 vs. 3)	ΔF: 21.4	1	0.96	0.99
2	Cho (2021)	251	Primary	ICDAS II	S vs.E, D (0 vs. 1–4)	ΔF: 7.8	0.88	0.82	0.94
						ΔR: 20.5	0.83	0.93	0.91
					S, IE vs. AE, D (0–2 vs. 3,4)	ΔF: 10.9	0.90	0.83	0.94
						ΔR: 22.5	0.89	0.76	0.92
3	Oh (2021)	177	Permanent	ADA classification & Bitewing	S&IE vs. AE&D (ADA 0,1 vs. 2,3)	ΔF: 59.9	0.76	0.74	0.84
						ΔR: 74.5	0.83	0.82	0.91
5	Jung (2018)	791	Permanent	ICDAS II	S vs. E&D (0 vs. 1–4)	ΔF: -	0.90	0.56	0.81
					S&IE vs. AE&D (0–2 vs. 3,4)	ΔF: -	0.91	0.84	0.93
					S&E vs. D (0–3 vs. 4)	ΔF: -	0.98	0.96	0.98
*Proximal*									
1	Oh (2022)	21	Permanent	Bitewing	E vs. D (1 vs. 2,3)	ΔF: 10.4	0.62	0.62	0.67
2	Cho (2021)	627	Primary	Bitewing ICDAS II	S vs. E, D (0 vs. 1–4)	ΔF: 7.8	0.83	0.74	0.87
					IE vs. AE, D (0–2 vs. 3,4)	ΔF: 9.2	0.91	0.74	0.91
3	Oh (2021)	91	Permanent	ADA classification Bitewing	S&E vs. D	ΔF: 6.0	0.74	0.73	0.81
						ΔR: 0.0	0.83	0.00	0.59
7	Kim (2017)	280	Permanent	Bitewing	S&E vs. D	ΔF: 12.4	0.83	0.82	0.86
						ΔR: 23.3	0.84	0.88	0.90

Se, sensitivity; Sp, specificity; AUC, area under the ROC curve; na, not available; n, number of teeth; S, sound tooth.

IE: incipient enamel caries (ICDAS score 1,2).

AE: advanced enamel caries (ICDAS score 3).

E: enamel caries (IE + AE, ICDAS score 1–3).

D: dentinal caries (ICDAS score 4).

**Table 3:** In vitro results.

Ref.	Author (year)	*n*	Dentition	Reference test	Diagnostic accuracy				
					Diagnostic threshold	Cut-off	Se	Sp	AUC
*Occlusal*									
4	Diniz (2019)	88	Primary	Histology	S vs. E&D	ΔF: >7.4	0.68	0.80	0.71
					S&E vs. D	ΔF: >13.8	0.93	0.87	0.88
8	Pontes (2017)	50	Primary	Histology	S vs. E&D	ΔF: >9.3	0.57	0.75	0.59
						ΔR: >21.9	0.72	0.75	0.67
					S&E vs. D	ΔF: >16.7	0.70	0.88	0.80
						ΔR: >31.3	0.90	0.83	0.87
12	Jallad (2015)	60	Permanent	Histology	S vs. E&D	ΔF: na	0.96	0.57	0.94
14	Gomez (2013)	112	Permanent	Histology	S vs. E&D	ΔF: na	0.72	0.91	0.97
					S&E vs. D	ΔF: na	0.86	0.81	0.92
15	Pereira, Eggertsson, González-Cabezas, Zero, Eckert and Mialhe (2011)	96	Permanent	Histology	S vs. E&D	ΔF: na	0.96	0.38	0.84
16	Zandoná, Santiago, Eckert, Fontana, Ando and Zero (2010)	400	Permanent	ICDAS	S vs. E&D	ΔF: na	0.82	0.92	na
*Proximal*									
6	Lee (2018)	62	Permanent	Histology	S vs. E&D	ΔF: 75	0.80	0.92	0.91
9	Yoon (2017)	102	Permanent	Radiograph	S vs. E&D	ΔF: 17.9	0.78	0.87	0.91
13	Ko (2015)	95	Permanent	Histology	S vs. E&D	ΔF: ≤13.8	0.75	0.84	0.80
					S&E vs. D	ΔF: >28.3	0.64	0.88	0.76
*Smooth*									
17	Shi (2001)	40	Permanent	Histology	S&E vs. D	na	0.83	0.98	na
						na	0.75	0.96	na

Se, sensitivity; Sp, specificity; AUC, area under the ROC curve; na, not available; n, number of teeth; S: sound tooth.

E: enamel caries.

D: dentin caries.

### Data synthesis

In the meta-analysis of in vivo studies, the pooled sensitivity and specificity for detecting occlusal caries were 0.86 and 0.82, respectively, while those for proximal caries were 0.74 and 0.82 (Table 4). In comparison, in vitro studies showed slightly lower values for occlusal caries, with a pooled sensitivity of 0.83 and specificity of 0.74. For proximal caries, the pooled sensitivity in the in vitro studies was slightly higher (0.83), whereas the specificity was lower (0.74) than in the in vivo data.

**Table 4:** Pooled sensitivity and specificity obtained from meta-analysis.

In vivo	Pooled sensitivity	Pooled specificity	In vitro	Pooled sensitivity	Pooled specificity
Occlusal (4 studies)	0.86	0.82	Occlusal (5 studies)	0.83	0.74
Permanent (3 studies)	0.86	0.80	Permanent (3 studies)	0.91	0.66
Primary (1 study)	–	–	Primary (2 studies)	0.63	0.81
Proximal (4 studies)	0.74	0.82	Proximal (3 studies)	0.83	0.74
Permanent (3 studies)	0.76	0.81	Permanent (3 studies)	–	–
Primary (1 study)	–	–	Primary (none)	–	–

**Fig. 3:**
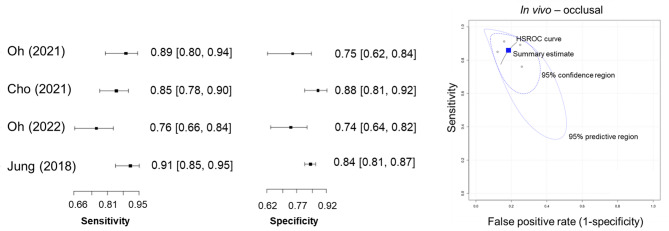
Forest plots and HSROC curve of diagnostic accuracy for detecting occlusal caries using QLF (in vivo studies).

Further analyses were conducted on studies that met the meta-analysis criteria. In vivo occlusal detection (four studies) demonstrated cumulative sensitivity and specificity of 0.86 and 0.82, respectively (Fig. 3). The HSROC curve showed a high overall summary estimate with a narrow 95% confidence interval and a relatively wide 95% predictive interval, reflecting consistent diagnostic performance with some variability across studies. For proximal caries, four studies were included in the meta-analysis, with a pooled sensitivity and specificity of 0.74 and 0.82, respectively. Subgroup analysis of permanent teeth (three studies) revealed a pooled sensitivity of 0.86 and specificity of 0.80 for occlusal caries, while proximal permanent teeth (three studies) showed a sensitivity of 0.76 and specificity of 0.81.

**Fig. 4:**
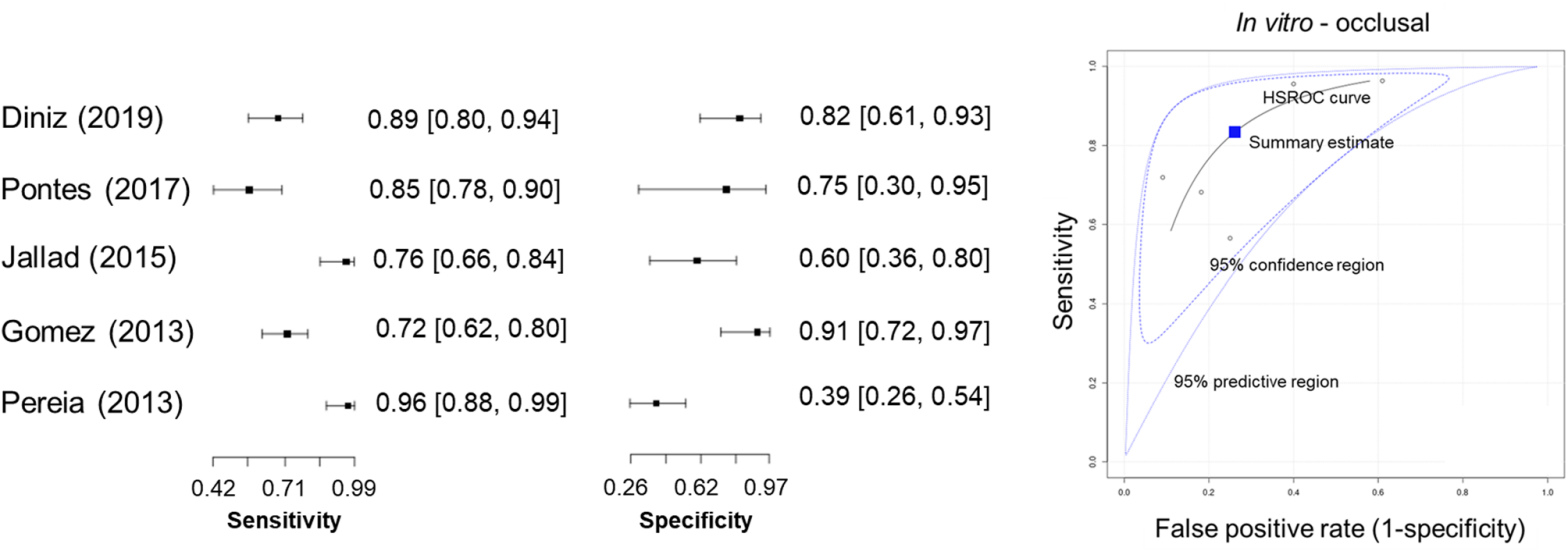
Forest plots and HSROC curve of diagnostic accuracy for detecting occlusal caries using QLF (in vitro studies).

Among the in vitro studies, five were included in the meta-analysis of occlusal caries detection. The HSROC curve (Fig. 4) presented a moderately narrow 95% confidence region and a wider 95% predictive region, reflecting the diagnostic variability among studies. Subgroup analysis showed pooled sensitivity and specificity of 0.91 and 0.66 for occlusal caries detection in the permanent dentition (three studies). For proximal caries in the permanent teeth (three studies), the pooled sensitivity and specificity were 0.83 and 0.74. Two studies on occlusal caries in primary teeth reported a pooled sensitivity and specificity of 0.63 and 0.81.

## Discussion

This systematic review and meta-analysis is the first to comprehensively evaluate the diagnostic accuracy of QLF technology for detecting dental caries across various lesion types. Seventeen studies were included, comprising 5 in vivo, 11 in vitro, and one study involving both environments. Quality assessment showed that most studies were of good quality, and the heterogeneity was moderate. Regardless of the study setting, tooth surface type, or dentition, the QLF demonstrated a consistent moderate-to-high diagnostic accuracy.

The meta-analysis revealed that QLF exhibited a particularly high diagnostic accuracy for early carious lesions and effectively distinguished between sound and demineralised enamel. In vivo studies showed superior sensitivity and specificity compared to in vitro studies, indicating consistent diagnostic performance. In contrast, in vitro studies demonstrated higher sensitivity but greater variability in specificity, potentially due to artificially varied lesion conditions and insufficient background homogeneity, which may have influenced the false-positive rates.

QLF has demonstrated significant utility in the detection of early occlusal lesions, supporting its applicability in clinical settings. Although in vivo studies have also shown a high diagnostic performance for proximal caries, the sensitivity was relatively lower than that for occlusal surfaces (pooled sensitivity 0.74), likely due to anatomical challenges in accessing proximal sites in the oral cavity^21,36^. However, the range of sensitivity for QLF in detecting proximal caries (0.62–0.91) surpassed that of traditional methods, including visual inspection (0.04–0.81), radiography (0.15–0.83), and DIAGNOdent (0.60–0.88), suggesting a comparative advantage^36^. A higher minimum value and narrower range of sensitivity indicated less inter-study variability and more stable diagnostic performance.

The high diagnostic accuracy of QLF is consistent with previous systematic reviews^15,37^. Heinrich‑Weltzien et al. (2005) reported that QLF detected early carious lesions more sensitively than visual inspection, with a detection rate of 87.2%^37^. Angmar‑Månsson and ten Bosch (2001) emphasized the quantitative reliability and reproducibility of QLF for detecting enamel demineralisation under both in vivo and in vitro conditions^15^. Gimenez et al. (2015) confirmed that fluorescence-based diagnosis, including QLF, maintained high sensitivity and diagnostic performance across different oral environments and tooth types^18^. Similarly, Twetman et al. (2012) reported that QLF outperformed traditional diagnostic tools such as visual-tactile inspection and radiography^38^. However, most of these earlier studies were based on laboratory conditions and lacked clinical applicability. A recent systematic review by Petros et al. (2021) included only two QLF studies, further limiting clinical conclusions^36^. This review addresses these gaps by incorporating more in vivo studies and conducting subgroup analyses according to lesion site and dentition type, thereby providing more robust evidence supporting the clinical utility of QLF. 

QLF showed high diagnostic accuracy not only for both dentin caries but also for early enamel lesions. Many in vivo studies targeted non-cavitated occlusal caries (ICDAS 1–2), with pooled sensitivity ranging from 0.88 to 0.90 when using ICDAS 1–2 as the diagnostic threshold. This is consistent with Gomez (2013), who found that the QLF showed superior sensitivity for detecting non-cavitated occlusal caries compared to other diagnostic tools, including visual inspection^39^. QLF enables detection of microstructural changes in dental hard tissues through biofluorescence, quantifying these changes via fluorescence loss (ΔF). This quantitative approach enables clinicians to promptly detect early lesions and implement preventive treatments to inhibit caries progression.

QLF utilises two core fluorescence indicators: ΔF, which reflects structural changes in demineralised hard tissue, and ΔR, which indicates red fluorescence from bacterial metabolites such as porphyrins. ΔF allows the quantitative assessment of lesion depth, whereas ΔR increases with lesion activity. Among the studies included, five specifically utilised ΔR as a diagnostic variable in this review reported high diagnostic accuracy for occlusal caries (AUC >0.9), even for incipient enamel lesions^20,40^. Combining ΔR and ΔF improved diagnostic performance, particularly in primary dentition^21^. This suggests that ΔR is not only an independent diagnostic marker but also a valuable supplement to ΔF in evaluating lesion presence and activity. Several studies support the utility of red fluorescence for assessing caries activity^41–43^, indicating that ΔR could help monitor lesion severity after preventive treatments. Although meta-analysis for ΔR was limited due to insufficient studies, future well-designed in vivo studies are expected to conclusively establish its clinical reliability.

The heterogeneity observed in this study likely stems from differences in the study design, diagnostic criteria, lesion characteristics, and caries prevalence. Among these, differences in QLF device type and cutoff values for ΔF were notable contributors. Earlier studies primarily used the first-generation QLF device (Inspektor Pro), whereas second generation devices such as QLF-D have been adopted since 2015, and third-generation intraoral camera-type devices such as Qraypen have been utilised in in vivo studies since 2020 onward^44^. Notably, second-generation devices introduced the ability to independently measure ΔR, enabling more quantitative assessment of lesion activity. With each successive generation, QLF devices have been optimised for clinical usability, resulting in variations in the device types across studies. Nevertheless, all devices are based on the same core QLF technology and produce ΔF values, which contribute to the consistent diagnostic accuracy across generations in terms of sensitivity, specificity, and AUC. This suggests that ΔF remains a reliable marker for caries detection and severity assessment, regardless of device configuration. Park et al. (2019) found no significant diagnostic differences between QLF generations for non-cavitated smooth surface caries, supporting the interchangeability of QLF devices in clinical settings. Although eligible studies for meta-analysis were limited, we minimised heterogeneity by stratifying the data by study design (in vivo vs. in vitro) and included only studies with comparable protocols. Additionally, the ΔF cutoff value can be flexibly adapted in clinical practice based on lesion depth, location, and dentition, serving as a practical tool for guiding treatment decisions rather than a rigid threshold.

Most of the included studies were assessed to be of good quality, particularly regarding the use of appropriate reference standards and consistent timing and flow, which enhanced the reliability of this meta-analysis. However, some studies lacked relevant clinical details, blinding protocols, or clearly defined patient selection criteria, which could have introduced potential bias. To strengthen the evidence base for QLF, future studies should address these limitations through standardised protocols and broader patient inclusion to improve its clinical applicability.

However, the clinical utility of QLF must be contextualized. The latest ORCA-EFCD consensus report maintains that visual examination is the primary diagnostic method^6^. This is consistent with findings from studies by Gomez et al. (2013) and Pontes et al. (2017), which concluded that visual inspection is often sufficient and that fluorescence-based methods may not offer a significant advantage for routine detection^19,32^. Therefore, the high diagnostic accuracy of QLF found in our meta-analysis does not suggest replacing visual examination. Instead, our findings support its role as a powerful adjunct tool for enhancing diagnostic confidence in uncertain cases, quantitatively monitoring lesion changes over time, and improving patient communication through real-time visualization.

The major strength of this review is its comprehensive evaluation of the diagnostic performance of QLF across a wide range of lesion types—from early enamel caries to cavitated lesions—using both in vivo and in vitro data. Subgroup analyses by study design, lesion location, and dentition type provided detailed insights into diagnostic accuracy and demonstrated the clinical applicability of QLF. Additionally, this review included studies that used recently developed devices such as Qraypen, to evaluate their practical utility. The QUADAS-2 tool was systematically applied for quality assessment to enhance the credibility of the results.

However, this study had some limitations. The number of studies included in the meta-analysis was limited (five in vitro and four in vivo studies), which reduced the statistical power. This may lead to a potential overrepresentation of individual study effects and limit the generalisability. Therefore, future research should aim to adopt standardised diagnostic protocols and include more diverse populations to evaluate the diagnostic utility of QLF more precisely.

Our findings highlight the clinical value of QLF, particularly for detecting incipient enamel caries (ICDAS codes 1 and 2) with high sensitivity and specificity. This supports preventive interventions at an early stage of the disease, potentially reducing the progression of caries and unnecessary treatment costs. Moreover, QLF enables monitoring of lesion changes using quantitative indicators—fluorescence loss (ΔF) and red fluorescence gain (ΔR)—which help clinicians make timely treatment decisions while minimising false positives.

To further validate the clinical utility of QLF, future studies should employ rigorous designs, particularly longitudinal studies that track early lesion detection and outcomes following preventive interventions. These studies are essential to assess the long-term cost-effectiveness and real-world benefits. In addition, multicenter studies across various populations—including children, older adults, orthodontic patients, and medically compromised patients, have been conducted across a range of clinical settings such as primary care clinics and community health centres.

Moreover, incorporating the QLF into epidemiological surveys and public health monitoring can help reduce inter-examiner variability and facilitate standardised, image-based diagnostics. QLF devices with digital imaging and transmission capabilities can contribute to effective disease surveillance and prevention strategies at the public health level and ultimately support the development of data-driven oral health policies.

## Conclusion

Based on this systematic review and meta-analysis, QLF demonstrates high diagnostic accuracy for detecting dental caries. Specifically, our analysis found the pooled in vivo sensitivity and specificity for occlusal caries to be 0.86 and 0.82, respectively, and 0.74 and 0.82 for proximal caries. The technology showed particular strength in identifying early, non-cavitated enamel lesions with high AUC values. However, considering the variability among studies and some evidence suggesting visual inspection remains sufficient in certain contexts, QLF cannot be recommended as a standalone diagnostic method. Its non-invasive and quantitative nature makes it particularly useful for objectively monitoring lesion progression over time and enhancing diagnostic confidence in clinically uncertain cases. Future well-designed, long-term clinical trials are warranted to establish standardized diagnostic thresholds and to verify the clinical reliability and cost-effectiveness of QLF technology across diverse populations.

## Materials and methods

This systematic review was conducted in accordance with the Preferred Reporting Items for Systematic Reviews and Meta-Analysis guidelines^45^. The review was designed to address the following structured research question based on the PICOS framework: “In individuals with dental caries lesions (Population), how accurate is the QLF diagnostic method (Intervention) in detecting caries compared with a reference standard (Comparison), in terms of diagnostic validity (Outcome)?”

### Literature search and selection strategies

A comprehensive literature search was performed using the Medline (Ovid) and Embase (Ovid) databases for articles published up to May 2023. Language restrictions were not imposed during the initial search. In addition, the reference lists of the included studies were manually screened to identify potentially relevant publications that might have been missed.

The search strategy combined Medical Subject Headings (MeSH) and keywords related to dental caries and QLF technology: (“dental caries/“ OR caries.mp. OR ([tooth OR teeth] AND (decays OR cavities OR carious OR demineralise OR remineralise). mp.) AND (“quantitative light-induced fluorescence”. mp. OR QLF.mp. OR “red fluorescence”. mp.)

Studies were deemed eligible for inclusion if they met the following criteria: (1) evaluated the diagnostic performance of QLF technology (i.e. sensitivity and specificity) to detect dental caries on occlusal, approximal, or smooth surfaces in human primary or permanent teeth; (2) employed a clearly defined and appropriate reference standard; (3) provided sufficient data to extract or calculate diagnostic accuracy measures, such as true positives (TP), false positives (FP), true negatives (TN), and false negatives (FN), or reported sensitivity and specificity values; and (4) were published in either English or Korean.

Two independent reviewers (JHH and HIJ) screened titles and abstracts for relevance, followed by a full-text review when eligibility was uncertain. Discrepancies between reviewers were resolved by a third reviewer (KBI). When multiple publications used the same dataset, only the most comprehensive or most recent version was included.

### Data extraction

For each of the selected articles, the following information was extracted: first author, publication year, sample size, number of examiners, study setting (in vitro or in vivo), type of dentition (primary or permanent), type of carious lesion (occlusal, approximal, or smooth), diagnostic validity measures (sensitivity and specificity area under the curve), and reference standard used. All eligible studies were included in the narrative synthesis, to the extent that data were available. A meta-analysis was conducted for studies that reported absolute values for TP, FP, TN, and FN, or provided sufficient data to infer these numbers in comparison to a reference standard. For studies with missing or incomplete data relevant to the meta-analysis, the corresponding authors were contacted to retrieve the necessary information. If a study reported diagnostic performance at multiple threshold levels, the optimal cutoff point, as defined by the original authors or yielding the highest combined sensitivity and specificity, was used. When multiple examiners were involved, the diagnostic values obtained from the most experienced examiner were included in the analysis.

### Risk of bias of individual studies

The quality of the included studies was assessed using the Quality Assessment of Diagnostic Accuracy Studies-2 (QUADAS-2) tool^46^, which is widely recommended for systematic reviews of diagnostic performance. This tool evaluates four domains: patient selection, index test, reference standard, and flow and timing. Each domain is assessed for risk of bias and concerns regarding applicability. Patient verification, appropriate reference standards, and applicability concerns were the main assessment points for this review. These domains were also considered possible sources of heterogeneity in the meta-analysis. We did not attempt to assign a numerical quality score or formally classify the studies. The risk of bias for each domain was categorised as low, high, or unclear, according to the QUADAS-2 criteria.

### Summary measures and synthesis of results

Enamel caries were categorised into two stages based on the ICDAS criteria: incipient enamel caries, defined as ICDAS codes 1 and 2, indicating the first visual change in the enamel or a distinct visual change seen when wet, and advanced enamel caries, defined as ICDAS code 3, characterised by localised enamel breakdown without dentin involvement. Dentin caries was defined as lesions with visible dentin involvement (ICDAS codes≥4). Each lesion category was compared against a reference standard either by histological or direct visual inspection. Qualitative synthesis was conducted separately for different diagnostic thresholds. Advanced enamel carious lesions (ICDAS 3) were compared with sound and incipient enamel caries (ICDAS 1, 2) in in vivo studies, and sound teeth were compared with both enamel and dentin caries in in vitro studies.

For quantitative synthesis, 2 × 2 contingency tables (TP, FP, TN, and FN) were extracted from eligible studies to calculate the pooled sensitivity, specificity, and area under the receiver operating characteristic curve (AUC). Meta-analyses were conducted using MetaDTA software. Summary estimates were derived using a hierarchical summary receiver operating characteristic (HSROC) model. HSROC curves were plotted to evaluate the consistency of the findings and assess asymmetry, which may indicate potential publication bias.

## Data Availability

The datasets used and/or analysed during the current study are available from the corresponding author on reasonable request.
